# The Lipase/Amylase Ratio (LAR) in Peripheral Blood Might Represent a Novel Prognostic Marker in Patients with Surgically Resectable Pancreatic Cancer

**DOI:** 10.3390/cancers12071798

**Published:** 2020-07-05

**Authors:** Michael Stotz, Dominik A. Barth, Jakob M. Riedl, Eva Asamer, Eva V. Klocker, Peter Kornprat, Georg C. Hutterer, Felix Prinz, Karoline Lackner, Herbert Stöger, Armin Gerger, Martin Pichler

**Affiliations:** 1 Division of Oncology, Department of Internal Medicine, Comprehensive Cancer Center Graz, Medical University of Graz, 8036 Graz, Austria; michael.stotz@medunigraz.at (M.S.); dominik.barth@medunigraz.at (D.A.B.); j.riedl@medunigraz.at (J.M.R.); eva_asamer@gmx.at (E.A.); eva.klocker@medunigraz.at (E.V.K.); felix.prinz@medunigraz.at (F.P.); herbert.stoeger@medunigraz.at (H.S.); armin.gerger@medunigraz.at (A.G.); 2 Division of General Surgery, Department of Surgery, Medical University of Graz, 8036 Graz, Austria; peter.kornprat@medunigraz.at; 3 Department of Urology, Medical University of Graz, 8036 Graz, Austria; georg.hutterer@medunigraz.at; 4 Institute of Pathology, Medical University of Graz, 8036 Graz, Austria; karoline.lackner@medunigraz.at; 5 Center for Biomarker Research in Medicine, Medical University of Graz, 8036 Graz, Austria

**Keywords:** amylase, lipase, pancreatic cancer, prognosis, surgical resection

## Abstract

Pancreatic enzymes might play a pivotal role in the pathophysiology and prognosis of pancreatic cancer. The aim of this study is to investigate the lipase/amylase ratio (LAR), representing a marker previously used in the differentiation of pancreatitis, as a potential prognostic marker in pancreatic cancer. Data from 157 surgically treated patients with ductal pancreatic adenocarcinoma and 351 patients with metastatic disease were evaluated retrospectively. Cancer-specific survival (CSS) was considered the endpoint of the study. After applying Kaplan–Meier curve analysis, uni- and multivariate Cox regression models were calculated to evaluate the prognostic relevance of LAR. An elevated LAR at diagnosis of localized pancreatic cancer was significantly associated with higher CA19-9 levels (*p* < 0.05). In univariate analysis, we observed an increased LAR as a significant factor for lower CSS in localized pancreatic cancer patients (HR = 1.63; 95% CI = 1.12–2.36; *p* = 0.01), but not in metastatic patients (HR = 1.12; 95% CI = 0.87–1.43; *p* = 0.363). In multivariate analysis, including age, gender, tumor stage, Karnofsky Performance Status, tumor grade, administration of chemotherapy and the LAR, an increased LAR was confirmed to represent an independent prognostic factor regarding CSS (HR = 1.81; 95% CI = 1.17–2.77; *p* = 0.007) in localized pancreatic cancer patients. In conclusion, our study identified the LAR as an independent prognostic factor in surgically treated pancreatic cancer patients.

## 1. Introduction

Despite tremendous advances regarding various treatment modalities over the recent years, pancreatic cancer remains to represent an exceptionally aggressive tumor entity with high mortality rates. Thus, notwithstanding intensive research efforts and the introduction of novel therapeutic agents, the prognosis of pancreatic cancer has only slightly improved over the last years [1]. This is for the most part due to a late onset of symptoms, usually resulting in undetected pronounced local tumor progression and the fast development of distant metastases. In consequence, an early and complete surgical tumor resection, representing de facto the only possibility of cure, is rarely achieved; however, at the time of the diagnosis only a small fraction of patients is considered to be surgically resectable [2,3]. Moreover, the lack of a fast administration of effective therapeutic regimens in most of the patients contributes to an overall poor survival rate in these cases. The application of chemotherapy at an early stage has slightly improved pancreatic cancer patient outcomes, albeit no breakthrough in terms of therapy has been achieved until now [4,5]. Neoadjuvant chemotherapeutic regimes, like FOLFIRINOX, represent valid options to accomplish a full surgical tumor resectability but in reality, often fail to reach their aim due to the high and limiting associated chemotherapy toxicities [6,7,8]. In the relatively rare cases of a curative tumor resection, the administration of adjuvant chemotherapy remains the gold standard in pancreatic cancer. Recommended therapeutic agents include gemcitabine or 5-FU/folinic acid for the duration of six months after surgery [9,10,11]. Recently, the ESPAC 4 randomized phase III trial, analyzing 730 resected pancreatic cancer patients, has been able to show a benefit of gemcitabine plus capecitabine, and recently the modified FOLFIRINOX regimen has entered the stage as a new adjuvant standard of care treatment. In contrast, the combination therapy of gemcitabine plus nab-paclitaxel failed to demonstrate a significant progression-free survival benefit in >860 resected pancreatic cancer patients in the APACT III trial [12].

The lack of efficient tumor therapy regimens, as well as the lack of robust prognostic factors for optimized and individualized treatment decisions in pancreatic cancer patients require intensified research efforts for potentially prognostic biomarkers regarding this complex tumor entity. On the one hand, histopathological factors, such as histological subtype, tumor grade, tumor size, vascular, perineural and lymphatic invasion, represent well-established prognostic parameters in pancreatic cancer [13,14]. On the other hand, reliable, ready-to-use peripheral blood-based molecular biomarkers in pancreatic cancer, that would be ideally easy to attain without laborious, cost-intensive and invasive procedures, are strongly warranted.

Over the recent years, various blood-based parameters of the systemic inflammatory response have been investigated, adding potential prognostic information in several cancer types [15,16,17,18,19,20]. Additionally, biochemical biomarkers, e.g., uric acid, were demonstrated to contain potentially important prognostic information in pancreatic cancer [21,22]. Both amylase and lipase have recently been investigated regarding their potential prognostic value in pancreatitis and pancreatic cancer, as well as in intraductal papillary mucinous neoplasm (IPMN) [23,24,25,26]. The lipase/amylase ratio (LAR) has been proposed as a possible new index that is able to distinguish acute episodes of alcoholic from non-alcoholic acute pancreatitis [27]. To the best of our knowledge, there are currently no data available regarding the LAR as a potential prognostic marker in pancreatic cancer. Therefore, the aim of the present study is to explore the potential role of the LAR regarding cancer-specific survival (CSS), assessed in peripheral blood at the time of the diagnosis, in a large cohort of patients with surgically resectable pancreatic cancer.

## 2. Materials and Methods

This retrospective analysis included data from 157 localized and 351 metastatic consecutive pancreatic cancer patients who were treated at the Department of Surgery and Division of Oncology, Department of Internal Medicine, Medical University of Graz, between 2004 and 2015. All patients had histologically confirmed ductal adenocarcinoma of the pancreas preoperative/pre-diagnostic lipase and amylase levels at the time of diagnosis were available in all reported patients. All patients with other histological subtypes of pancreatic cancer were excluded from analyses. All clinico-pathological patient data were obtained from medical records of our institution (Division of Oncology, Department of Internal Medicine, Medical University of Graz) and from pathology records from the Institute of Pathology. Since the TNM classification system for pancreatic cancer underwent changes during the study period, tumor stages were uniformly adjusted according to the 7th edition of the TNM classification system. Parameters included in the LAR that further documented clinico-pathological data included the administration of chemotherapy with gemcitabine, as well as age and gender of the patients included in the study. Chemotherapy was administered in patients who were considered fit for chemotherapy according to age, performance status or any contraindication/patient’s wish. The peripheral blood-based lipase and amylase levels were obtained within one to seven days before histologically proven diagnosis/any interventional procedure and assessed as a pre-intervention routine procedure. The LAR was calculated as the amount of lipase divided by the amount of amylase in units per liter, whereby the analysis of these amounts was performed in the general routine laboratory of the Medical University of Graz using an automated analyzer (Sysmex Corporation, Kobe, Japan). Follow-up evaluations were performed every three months within the first three years, every six months for five years and annually thereafter for curatively resected tumor stages. Clinical check-ups, laboratory tests (including carcinoembryonic antigen CEA and CA19-9) and radiological assessments where performed in the follow-up period in all cases. We obtained dates of death, which were from the central registry of the Austrian Bureau of Statistics, whereby patients’ records/information were anonymized and de-identified prior to analysis. This study was approved by the local ethical committee of the Medical University of Graz (No. 26-196 ex 13/14).

Cancer-specific survival (CSS) was defined as the time (in months) from the date of surgery or date of histologically proven diagnosis to cancer-related death. The optimal cut-off value for the preoperative LAR was determined by applying receiver operating curve analysis (ROC) to differentiate between patients’ survival and death (using the MedCalc statistical software version 13.3, MedCalc Software Ltd., Ostend, Belgium). The relationship and correlation between the pancreatic enzymes and clinico-pathological parameters were assessed by non-parametric tests (Mann–Whitney U and chi square test). Patients’ clinical endpoint was calculated using the Kaplan–Meier method and differences between prognostic groups were compared by the log-rank test. Backward stepwise multivariate Cox proportion analysis was implemented to determine the influence of different clinico-pathological parameters and the LAR on CSS, whereas age, gender, Karnofsky index (KI), tumor stage, administration of chemotherapy, CA19-9 levels and LAR were included in the multivariate model. Hazard ratios (HRs) were reported as relative risks with corresponding 95% confidence intervals [28]. All statistical analyses were performed using the Statistical Package for Social Sciences version 20.0 (SPSS Inc., Chicago, IL, USA). We considered a two-sided *p* < 0.05 as statistically significant.

## 3. Results

Overall, 76 male and 81 female pancreatic cancer patients were included into analyses. The average age at diagnosis was 64 ± 9.4 yrs. Median survival was 16 months (range: 0–112 months) and at the time of analyses 118 out of all patients had died by their most recent follow-up. Adjuvant chemotherapy was administered to 121/157 (77%) patients. Low grade (G1 + G2) tumors were more frequent in 92 (59%) patients than high grade (G3 + G4) carcinomas in 65 (41%) patients. Out of the 157 patients, the majority had stage IIb (*n* = 100) and IIa (*n* = 29) tumors, whereas the minority had stage I (*n* = 12) and stage III (*n* = 16) tumors. A Karnofsky index (KI) ≥ 90 was observed in 78 (50%) patients, whereas the other half of the patient cohort had a lower KI (KI 80% *n* = 51, KI < 80% *n* = 28). Lipase and amylase levels were obtained from all 157 patients. The median amylase level was 25 U/L (interquartile range: 13 to 43, range: 0–664 U/L) and the median lipase level was 47 U/L (interquartile range: 25 to 92, range: 6–1962 U/L). Applying ROC analysis, we calculated an optimal cut-off level for amylase being 56, for lipase being 9 and the preoperative LAR being 2.74 (Youden index 0.2188). For the LAR, the area under the ROC curve (AUC) was 0.605 (95% CI: 0.524 to 0.682), with a sensitivity of 39.83 and a specificity of 82.05.

In the cohort studied, we were able to demonstrate a statistically significant association between the preoperative LAR and CA19-9 levels, whereas no association between the preoperative LAR and patient age, gender, the administration of chemotherapy, tumor stage and tumor grade was observed (see Table 1). In 94 patients with available information on tumor localization (caput versus non-caput), no significant association between the LAR was identified (*p* > 0.05).

**Figure 1:**
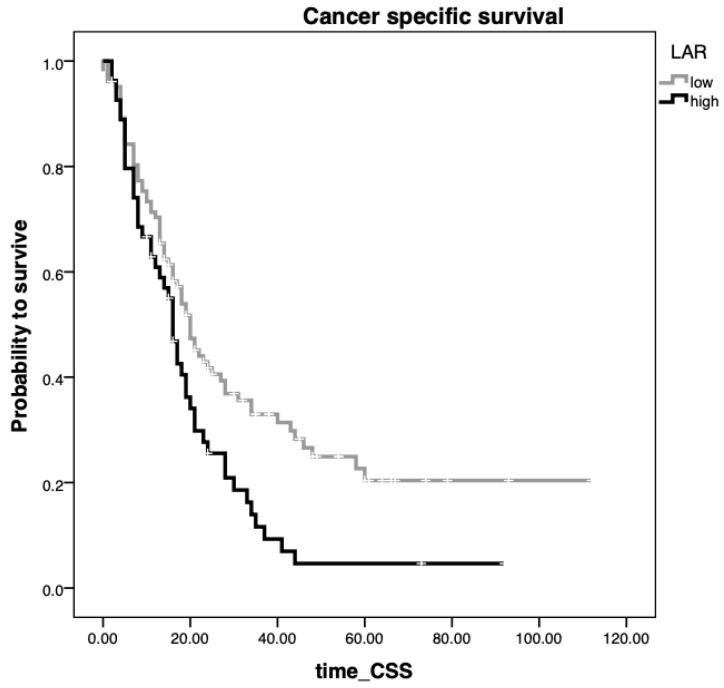
Kaplan–Meier curves predicting cancer-specific survival (CSS) regarding a preoperative LAR < 2.74 vs. LAR ≥ 2.74 in the study cohort comprising 157 patients undergoing surgical resection (*p* = 0.008).

Figure 1 shows the Kaplan–Meier curves regarding CSS and reveals that a preoperative LAR > 2.74 represents a consistent factor for poor prognosis in pancreatic cancer patients undergoing surgical resection (*p* = 0.008, log-rank test).

To investigate whether the preoperative LAR and other clinico-pathological parameters are associated with the clinical outcome of pancreatic cancer patients, univariate and multivariate Cox regression models regarding patient CSS were calculated. Univariate analysis identified the tumor stage (<0.001), CA19-9 level (elevated or not, *p* = 0.009), the administration of chemotherapy (chemotherapy vs. no treatment, *p* = 0.006) and the preoperative LAR (<2.74, ≥2.74, *p* = 0.01) as poor prognostic factors regarding CSS in this study cohort. High tumor grade, age, gender, as well as the amylase and the lipase level were not statistically significantly associated with clinical outcome (Table 2).

**Figure 2:**
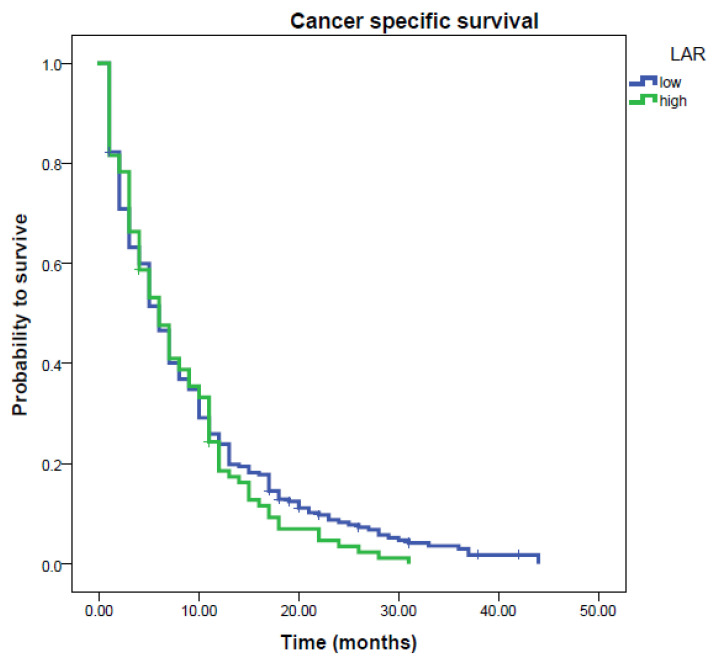
Kaplan–Meier curves predicting cancer-specific survival (CSS) regarding a pre-diagnostic LAR < 1.33 vs. LAR ≥ 1.33 in the study cohort comprising 351 metastatic non-surgical resectable PDAC patients (*p* = 0.335).

To determine the independent prognostic value of the preoperative LAR regarding CSS, a multivariate analysis using a Cox proportional hazard model was performed. In the multivariate analysis including age, gender, CA19-9 level, tumor stage, application of chemotherapy, KI and the preoperative LAR, we identified high tumor stage, the administration of chemotherapy, KI and the preoperative LAR to represent independent prognostic factors regarding CSS (HR = 1.63; 95% CI = 1.17–2.77; *p* = 0.007; Table 2). To expand our study and test the hypothesis whether the LAR represents a prognostic factor in metastatic non-surgical resectable pancreatic ductal adenocarcinoma (PDAC), we analyzed 351 metastatic PDAC cases, the median LAR was 2.05 (interquartile range: 1.42–2.89, minimum: 0.36, maximum: 25.67). Overall, 199 male and 152 female patients were included into analyses. The average age at diagnosis was 65 ± 10.1 yrs. Median survival was 6 months (range: 0–44 months) and at the time of analyses 338 (96.3%) out of all patients had died by their most recent follow-up. Chemotherapy was administered to 246/351 (71%) patients. Low grade (G1 + G2) tumors were more frequent in 221 (59%) patients than high grade (G3 + G4) carcinomas in 143 (41%) patients. A Karnofsky index (KI) ≥ 90 was observed in 221 (63%) patients and a lower performance status of KI < 90% was observed in 130 (37%) of patients. Lipase and amylase levels were obtained from all 157 patients. The median amylase level was 21 U/L (interquartile range: 12 to 41, range: 1–561 U/L) and the median lipase level was 37 U/L (interquartile range: 22 to 79, range: 5–1597 U/L). Applying ROC analysis, we calculated an optimal cut-off level for the LAR being 1.33 (Youden index 0.2518). For the LAR, the area under the ROC curve (AUC) was 0.587 (95% CI: 0.533 to 0.640), with a sensitivity of 79.03 and a specificity of 46.15. Applying the Kaplan–Meier curve method showed no survival difference between low and high LAR groups in metastatic PADC patients (log-rank test *p* = 0.335, Figure 2).

Univariate Cox proportional analysis shows no significant difference for survival for the LAR (HR: 1.12, 95% CI: 0.87–1.43, *p* = 0.363), whereas—as previously reported [26]—for amylase (HR: 1.26, 95% CI: 10.01–1.57, *p* = 0.039) and lipase (HR: 1.25, 95% CI: 1.01–1.56, *p* = 0.039) a significant association with survival could be observed.

## 4. Discussion

In the present study, we examined a large cohort of patients with surgically resected pancreatic cancer and found a statistically significant association between the preoperative LAR in the peripheral blood and the patients’ survival. In a recently published study, our group reported the value of amylase levels as an adverse factor in metastatic pancreatic cancer [26]. In this cohort of localized pancreatic cancer patients, we could not find an association between amylase or lipase levels and survival. However, to the best of our knowledge this is the first study investigating the potential prognostic impact of the preoperative LAR in localized pancreatic cancer.

The pancreatic triglyceride lipase represents one of several lipases secreted by the exocrine pancreas, whereby its important role in fat digestion is evident, as patients with an isolated deficiency of this lipase suffer from fat malabsorption [29]. Moreover, pancreatic triglyceride lipase is a carboxyl esterase with a strong preference for acylglycerides over phospholipids, cholesterol esters, and galactolipids [30,31]. Since the pancreatic triglyceride lipase concentration in pancreatic tissue is high, it might be hypothesized that an injury of the pancreas leads to an increased serum activity due to cell leakage and an enzyme transfer into peripheral blood. Hence, in the case of acute pancreatitis, elevated lipase levels might be explained by the release of enzymes into the peripheral blood. In the case of pancreatic cancer, it is imaginable that similar tissue destruction might lead to changes in peripheral lipase levels caused by a tumorous infiltration of pancreatic tissue. On the other hand, low peripheral lipase levels might result from an advanced loss of normal pancreatic cells as a result of tumor progression, comparable to lowered enzyme levels in the case of an organ insufficiency, e.g., in the case of chronic pancreatitis [32].

The pancreas and the salivary glands represent the main production sites of amylase, an enzyme with several isoforms, whose main function it is to break down starch into smaller polysaccharides at the internal 1 to 4 alpha linkage during the digestive process [33]. However, amylase can also be found in smaller quantities in other tissue types [34]. An elevation of peripheral amylase levels might occur in a variety of different diseases. Acute and chronic pancreatitis, renal failure and renal clearance impaction, also alcohol abuse or cholestatic disorders can lead to an elevation of amylase levels. Thus, the potential prognostic value of amylase, as well as lipase, might be quite poor and is corroborated by our results, since we found no statistically significant association between these enzyme levels and CSS in pancreatic cancer patients (data not shown).

Elevated levels of serum amylase and/or lipase are used either alone or in combination in many healthcare centers for the diagnosis of acute pancreatitis, however, without acknowledging which one could provide a better diagnostic performance. A recent review by Ismail et al. has showed that serum lipase might offer a higher sensitivity than serum amylase in diagnosing acute pancreatitis [35]. However, there is a scarcity of data indicating that a measurement of either lipase or amylase can be used as a good diagnostic or prognostic tool in pancreatic cancer patients. Gultepe and colleagues have recently shown that lowered lipase levels close to zero might represent a potential indicator for pancreatic cancer [24]. Ventrucci et al. found a highly variable enzyme behavior with low serum lipase levels (10%) and high serum lipase levels (25%) in patients with pancreatic cancer—the authors thus concluded that pancreatic enzymes might have no added diagnostic value, neither for chronic pancreatitis nor pancreatic cancer patients [36,37]. This phenomenon is corroborated by our own results, since neither the preoperative amylase, nor lipase levels were significantly associated with patients’ CSS.

Nonetheless, we tested the index of preoperative LAR, which had already been investigated nearly three decades ago in acute pancreatitis. Interestingly, an elevated preoperative LAR was significantly associated with a poorer CSS. Currently, there are no published data about the exact role of the LAR in pancreatic cancer and only sparse and moreover conflicting data about its role in acute pancreatitis are known. Some reports have suggested that the LAR might be useful for the differentiation between alcoholic and non-alcoholic pancreatitis; however, these observations were not validated in later studies [27,38,39,40]. Therefore, we can only speculate about the pathophysiological reasons for our finding. Since a great majority of our patients were treated surgically due to a low tumor stage, it might be hypothesized that at this point of time in the tumor disease, a lipase elevation by tumor destruction is greater than amylase’s. Based on our findings, the non-invasive measurement of preoperative amylase and lipase levels and the calculation of the LAR at the time of localized pancreatic cancer diagnosis might represent a novel marker for individualized patient risk assessment in surgically resectable pancreatic cancer.

Interestingly, we did not observe a prognostic value for the LAR in patients with metastatic PDAC. The reason for this observation remains speculative, but may include a different setting of underlying biology or treatment modality. In localized PDAC, surgery is the utmost important step for cure, whereas in metastatic PDAC, the application of chemotherapy with all mechanisms behind—efficacy, toxicity and resistance—is a critical factor for survival times.

A strength of our study is the sample size and a relatively long follow-up period. Limitations include of course the single-center retrospective design, which is responsible for a potential selection bias in our study cohort. As with all single center studies, our study lacks external validation, which at least should be done in future approaches.

## 5. Conclusions

Based on our results, the LAR may represent a novel prognostic biomarker that could potentially influence clinical decision making.

## Figures and Tables

**Table 1:** The relationship between clinico-pathological parameters and the pretreatment lipase-to-amylase ratio (LAR) in patients with surgically resectable pancreatic adenocarcinoma (*n* = 157).

Characteristics	LAR < 2.74	LAR ≥ 2.74	*p*-Value
Age at operation (y)			
<65	52	28	0.871
≥65	51	26	
Gender			
Female	52	29	0.701
Male	52	25	
CA19-9 levels			
<median	71	28	0.011
>median	19	20	
Tumor stage			
Stage I–II	88	43	0.372
Stage III	15	11	
Tumor grade			
G1 + G2	61	31	0.826
G3 + G4	42	23	
Chemotherapy			
No	24	12	0.879
Yes	79	42	

**Table 2:** Univariate and multivariate Cox regression analyses regarding cancer-specific survival in the study cohort of surgically resectable pancreatic adenocarcinoma patients (*n* = 157).

Parameter	Univariate Analysis		Multivariate Analysis	
	HR (95% CI)	*p*-Value	HR (95% CI)	*p*-Value
Age at operation				
continuously coded	1.02 (1.0–1.04)	0.067	1.0 (0.98–1.03)	0.589
Gender				
Female	1 (reference)	0.756	1 (reference)	0.888
Male	1.06 (0.74–1.52)		1.03 (0.68–1.55)	
Tumor stage				
Stage I-II	1 (reference)		1 (reference)	
Stage III	1.50 (1.28–1.77)	<0.001	3.52 (2.09–5.91)	<0.001
Tumor grade				
G1 + G2	1 (reference)	0.194	n.a.	
G3 + G4	1.27 (0.88–1.84)			
Chemotherapy				
No	1 (reference)	0.006	1 (reference)	0.020
Yes	0.54 (0.34–0.84)		0.52 (0.30–0.90)	
CA19-9 elevated				
No	1 (reference)	0.009	1 (reference)	0.075
Yes	1.72 (1.14–2.59)		1.50 (0.96–2.34)	
LAR				
<2.74	1 (reference)	0.010	1 (reference)	0.007
≥2.74	1.63 (1.12–2.36)		1.81 (1.17–2.77)	
Karnofsky index				
≤80	1 (reference)	0.029	1 (reference)	0.032
90–100	0.66 (0.46–0.95)		0.63 (0.41–0.96)	
Amylase elevated				
No	1 (reference)	0.654	n.a.	
Yes	0.90 (0.57–1.42)			
Lipase elevated				
No	1 (reference)	0.176	n.a.	
Yes	0.57 (0.25–1.29)			
